# BDS/GPS Dual Systems Positioning Based on the Modified SR-UKF Algorithm

**DOI:** 10.3390/s16050635

**Published:** 2016-05-03

**Authors:** JaeHyok Kong, Xuchu Mao, Shaoyuan Li

**Affiliations:** School of Electronic Information and Electric Engineering, Shanghai JiaoTong University, 800 Dongchuan Street, Minhang District, Shanghai 200240, China; jhkong2014@163.com (J.K.); syli@sjtu.edu.cn (S.L.)

**Keywords:** Global Navigation Satellite System (GNSS), positioning algorithm, modified square-root Unscented Kalman filter (modified SR-UKF), BeiDou navigation System (BDS)

## Abstract

The Global Navigation Satellite System can provide all-day three-dimensional position and speed information. Currently, only using the single navigation system cannot satisfy the requirements of the system’s reliability and integrity. In order to improve the reliability and stability of the satellite navigation system, the positioning method by BDS and GPS navigation system is presented, the measurement model and the state model are described. Furthermore, the modified square-root Unscented Kalman Filter (SR-UKF) algorithm is employed in BDS and GPS conditions, and analysis of single system/multi-system positioning has been carried out, respectively. The experimental results are compared with the traditional estimation results, which show that the proposed method can perform highly-precise positioning. Especially when the number of satellites is not adequate enough, the proposed method combine BDS and GPS systems to achieve a higher positioning precision.

## 1. Introduction

With the development of space information technology, some countries are constructing Global Navigation Satellite Systems (GNSS). Now, in addition to USA’s GPS and Russia’s GLONASS, Europe’s Galileo and China’s BeiDou navigation satellite System (BDS) are being built. Japan, India and other countries are also planning to build their own regional navigation satellite systems. Even though it is GPS that is the most developed navigation satellite system and it has many advantages, it also has some disadvantages of system reliability that cannot satisfy the requirements of a single navigation system in certain situations [1]. Recently, the idea of multi-navigation positioning that consists of GPS, GLONASS, Galileo and regional satellite positioning system is gradually getting more interest in the field of satellite navigation. Especially, the combination of GPS and BDS can overcome the deficiency of the single system, and shows better effects on system performance [2].

The most conventional positioning estimation method is the iterative least square method (ILS). Furthermore, extended Kalman filter (EKF) and unscented Kalman filter (UKF) are also used to estimate the positioning data. ILS can solve three-dimensional positioning only when it receives the signals from at least four satellites. This method is simple, and its computing speed is fast, but it has a large linearization error and a low positioning estimation precision. The EKF can only be accurate for a first order Taylor series. There may be a larger nonlinear error, and it needs to compute the Jacobian matrix, in addition to the calculation being difficult and one of the main sources of error [3]. The UKF represents statistical properties of the system by deterministic sampling and avoids the disadvantage that the EKF must compute the Jacobian matrix. Theory shows that the EKF predicts the means correctly up to the second order of Taylor series and covariances up to fourth order. In contrast, the UKF predicts the means and covariances correctly up to the fourth order [4,5].

Currently, unscented Kalman filter (UKF) and square root UKF (SR-UKF) are the widely used nonlinear filtering strategies, their applications are proposed. In the process of filtering, the calculation error exists. The accumulation of the calculation error reduces the filtering precision, and even it can make the error covariance matrices gradually lose their positive semidefiniteness [6]. In order to improve the numerical performance, the SR-UKF was proposed [7]. Here, Cholesky factors of the covariance matrices are directly used to calculate the state estimate, the QR decomposition, as well as Cholesky factor updates. Thus, by this way, the numerical stability can be improved and also the positive semi-definiteness of covariance matrices can usually be guaranteed [8].

In practical applications, the statistic models of the extended noises are difficult to build. In this case, Kalman filter will be invalid, so the adaptive filtering and robust filtering theory is brought and developed [9].

Because the norm of estimation error covariance decreases progressively in the process of filtering, the effects of new observations data for improving state estimation will be weakened. In fact, the changes of system dynamic model are difficult to fully know in advance. As the recent observation data contains more information about the changed system model, a modified SR-UKF algorithm is proposed in order to increase the weight of the new measurement data.

This paper proposes a BDS-GPS system model, using the modified SR-UKF algorithm to perform position estimation, and the experimental results illustrate the effectiveness of our proposed method.

Section 2 lists the general concept of the GNSS positioning. Section 3 introduces the modified SR-UKF algorithm which will be used in our proposed models for GNSS position estimation. Section 4 describes the unification of the reference systems and the time systems of GPS and BDS. Section 5 addresses the developed nonlinear model and the filter implementation . Section 6 includes recent experimental results and provides the comparison of these results from GPS and BDS with ILS, UKF, SR-UKF and the proposed method. Finally, Section 7 summarizes this paper, and puts forward the future developments.

## 2. GNSS Positioning Overview

GNSS is a worldwide all-weather navigation system which can provide tridimensional position, velocity, and time synchronization to the UTC scale. GNSS considers the earth’s center as the reference point, to determine the position of the receiver antenna in the reference coordinate system. Since the positioning operation requires only one receiver, it is called standalone positioning. The basic principle of the standalone GNSS positioning is taking the observed distance between the GNSS satellite and the user receiver antenna as the benchmark, which is based on the known instantaneous satellites’ coordinates, to determine the position of the corresponding user receiver antenna. According to the different positions of the user receiver antennas, GNSS positioning can be divided into dynamic positioning and static positioning. Currently, GNSS positioning has a variety of modes, such as precise point positioning (PPP) and relative positioning. Precise point positioning uses the precise ephemeris and satellite clock bias data provided from the International GNSS Service (IGS) and calculates the precise user coordinates from the corrected carrier phase and pseudorange. The relative positioning uses single or double difference of the carrier phase, and calculates the relative coordinates comparing to one or several base stations. These technologies can perform high precise positioning, but they need some accessories besides the user GNSS receiver, such as radio, network equipment, and the other GNSS receivers. In a word, they are not pure standalone positioning, due to their high cost and complex setup, and they are unsuited to the daily applications such as car navigation.

GNSS positioning is based on the one-way ranging technique: the propagation time to transmit from satellite to user receiver is measured and multiplied by the signal propagation velocity to obtain satellite-to-user range. The offset of the receiver clock relative to the system time scale should be estimated to position. The measured range between receiver and satellite is referred to as pseudorange, and can be represented as follows:

(1) $$\begin{array}{ccc} \;\rho_{i} & = & r_{i}+c\delta t_{u}+\varepsilon_{i} \end{array}$$

where $\rho_{i}$ is the *i*th satellite’s pseudorange measurement, $r_{i}$ is the geometric distance receiver-satellite, $c\delta t_{u}$ is the receiver clock offset (scaled by speed of light *c* ), and $\varepsilon_{i}$ contains the residual errors after satellite-based and atmospheric error corrections [10].

Equation (1) is applicable to the single GNSS (*i.e.*, BDS or GPS only), it contains the time scale of the considered system. However, for the multiple constellation case, another unknown variable used to represent the inter-system offset should be further estimated.

## 3. The Modified SR-UKF Algorithm

The UKF algorithm is the minimum variance estimation based on UT (Unscented Transform). It was first proposed by Julier *et al.* [11] in 1995. The state distribution here is represented by a number of appropriately chosen points, which is different from the Gaussian Random Variables (GRV) in UKF with deterministic samplings. These points evolve according to the dynamics of the true nonlinear system. Hence, compared with the EKF, the UKF not only has the possibility to improve the estimate precision, but also is easier to be implemented. Moreover, different from EKF, the evaluations of the Jacobian and any order of partial derivatives are not needed in the UKF. Some papers proposed the new EKF and UKF algorithm [12,13,14,15], and were used in GPS positioning [16,17,18,19,20]. The fuzzy adaptive UKF algorithm was applied in spacecraft celestial navigation [21]. When no more than four satellites can be received, a precision of data processing can be obtained by considering the UKF algorithm’s small linearization error.

The UKF is mainly used for an arbitrary nonlinear system, and numerical instability often causes the covariance matrix *P* to lose its positive definiteness during the filtering procedure. Consequently, the sigma points ${\hat{x}}_{t-1}\pm \sqrt{\left(L+\lambda \right)P_{t-1}}$ cannot be correctly calculated, where ${\hat{x}}_{t-1}$ is a priori estimate of state, $P_{t-1}$ is a priori covariance matrix of state, (for *L* and *λ*, see Equation (5)). Moreover, in the UKF design, the demanding operation is the evaluation of the square root of the covariance matrices at each time instant for the updated set of sigma points. To solve this problem, the SR-UKF was proposed [7].

Meanwhile, in the application of positioning, the exact knowledge of the noise matrix which is required in the framework of the Kalman filter is usually unknown and time-varying in practice. The inappropriate prior statistics in the Kalman filter cause large estimation errors or even errors possibly diverging. Because of the uncertain process noise, the standard UKF yields poor performance in robustness and tracking accuracy.

In the process of standard filtering, the norm of the estimation error covariance matrix is reduced with time, thus the effects of observations for correction of the state estimation are more and more weakened. As the recent observations contain more information on the changed dynamic system model, in the process, the effects of new observations for the state estimation error must be enhanced, and the effects of the old observations must be reduced.

First, assume that state and measurement equations of the system are discrete time nonlinear systems:

(2) $$\left\{\begin{array}{c} \;x_{t+1}=f\left(x_{t},w_{t}\right)\\ \;z_{t}=h\left(x_{t}\right)+v_{t} \end{array}\right.$$

where $x_{t}$ is state vector, $z_{t}$ is measurement vector, and $w_{t}$ is zero-mean independent Gaussian white noise, of which the covariance matrix is *Q*. $v_{t}$ is zero-mean independent Gaussian white noise of the measurement, of which the covariance matrix is *R*.

The UKF and SR-UKF algorithms are given in Table 1 and Table 2.

Details about the modified SR-UKF algorithm are described as follows:

Initialize with

(3) $$\begin{array}{cc} & {\hat{x}}_{0}=E\left[x_{0}\right]\\ & S_{0}=chol\left\{E\left[\left(x_{0}-{\hat{x}}_{0}\right){\left(x_{0}-{\hat{x}}_{0}\right)}^{T}\right]\right\} \end{array}$$

where chol{·} denotes Cholesky factorization. For a positive definite matrix *A*, if a matrix *X* is lower triangularly denoted by $A=XX^{T}$, then *X* is the Cholesky factor of *A*. The shorthand notation chol{·} denotes a Cholesky factorization, namely, X=chol{A} .

**Calculate the sigma points:**

(4) $$\begin{array}{c} \chi_{t-1}=\left[{\hat{x}}_{t-1}\;\;\;{\hat{x}}_{t-1}\pm \sqrt{\left(L+\lambda \right)}S_{t-1}\right] \end{array}$$

**Calculate weight coefficient:**

(5) $$\begin{array}{ccc} W_{0}^{\left(m\right)} & = & \lambda /(L+\lambda )\\ W_{0}^{\left(c\right)} & = & \lambda /\left(L+\lambda \right)+\left(1-\xi^{2}+\eta \right)\\ W_{i}^{\left(m\right)} & = & W_{i}^{\left(c\right)}=1/\left[2\left(L+\lambda \right)\right]\;\;i=1,\cdots ,2L \end{array}$$

where $\lambda =\xi^{2}\left(L+k\right)-L$ is a scaling parameter. The constant *ξ* determines the spread of the sigma points around a mean of state *x*, and is usually set to a small positive value (e.g., 0≤ξ≤1/2). The constant *k* is a secondary scaling parameter, which is usually set to 3-L, and *η* is used to incorporate prior knowledge of the distribution of *x* (for Gaussian distributions, η=2 is optimal) [5,11].

**Time-update equations:**

(6) $$\begin{array}{cc} \chi_{t|t-1}^{*}= & f(\chi_{t-1})\\ {\hat{x}}_{t|t-1}= & \sum_{i=0}^{2L}W_{i}^{\left(m\right)}\chi_{i,t|t-1}^{*} \end{array}$$

(7) $$S_{t|t-1}=qr\left\{\sqrt{W_{1}^{\left(c\right)}}\left(\chi_{1:2n,t|t-1}^{*}-{\hat{x}}_{t|t-1}\right)\;\;\;\sqrt{Q}\right\}$$

(8) $$S_{t|t-1}=S^{-1}cholupdate\left\{S_{t|t-1},\chi_{0,t|t-1}^{*}-{\hat{x}}_{t|t-1},W_{0}^{\left(c\right)}\right\}$$

(9) $$\begin{array}{c} \chi_{t|t-1}=\left[\chi_{0,t|t-1}^{*}\;\;\;\chi_{0,t|t-1}^{*}\pm \sqrt{\left(L+\lambda \right)}S_{t|t-1}\right] \end{array}$$

(10) $$z_{t|t-1}=h\left(\chi_{t|t-1}\right)$$

(11) $${\hat{z}}_{t|t-1}=\sum_{i=0}^{2L}W_{i}^{\left(m\right)}z_{i,t|t-1}$$

where qr{·} denotes QR decomposition. A matrix $A\in {ℜ}^{l\times n}\left(n\geq 1\right)$ is given by $A^{T}=QR$, and $Q\in {ℜ}^{n\times n}$ is orthogonal, and $R\in {ℜ}^{l\times n}$ is upper triangular. $\tilde{R}$ is the upper triangular part of *R*. We use the shorthand notation qr{·} to denote QR decomposition, namely, $\tilde{R}=qr\left\{A^{T}\right\}$, ${}^{\prime }cholupdate$ is the update to Cholesky factorization, cholupdate(R,X,ε) returns the Cholesky factor of $A+\varepsilon XX^{T}$, where *R* is the original Cholesky factorization of *A*.

**Measurement-update equations:**

(12) $$S_{z_{t}}=qr\left\{\sqrt{W_{1}^{\left(c\right)}}\left(z_{1:2n,t|t-1}-{\hat{z}}_{t|t-1}\right)\;\;\;\sqrt{R_{t}}\right\}$$

(13) $$S_{z_{t}}=cholupdate\left\{S_{z_{t}},z_{0,t|t-1}-{\hat{z}}_{t|t-1}W_{0}^{\left(c\right)}\right\}$$

(14) $$\begin{array}{c} P_{x_{t}z_{t}}=\sum_{i=0}^{2L}W_{i}^{\left(c\right)}\left[\chi_{i,t|t-1}-{\hat{x}}_{t|t-1}\right]\cdot {\left[z_{i,t|t-1}-{\hat{z}}_{t|t-1}\right]}^{T} \end{array}$$

(15) $$K_{t}=\left(P_{x_{t}z_{t}}/S_{z_{t}}^{T}\right)/S_{z_{t}}$$

(16) $${\hat{x}}_{t}={\hat{x}}_{t|t-1}+K_{t}\left(z_{t}-{\hat{z}}_{t|t-1}\right)$$

(17) $$U=K_{t}S_{z_{t}}$$

(18) $$S_{t}=cholupdate\left\{S_{t|t-1},U,-1\right\}$$

where,

(19) $$R_{t}=SR_{t-1}$$

and *S* is selected by experience. If *S* is too large, it will cause filter oscillation. Thus, generally, S is chosen to be slightly greater than one. (In this experiment, S=1.001.)

## 4. The Unification of Time and Reference Systems of BDS and GPS

### 4.1. The Unification of the Two Reference Systems

For the reference systems of BDS’s CGCS2000 and GPS’s WGS84, the definitions are essentially the same [22], and the reference ellipsoids are very similar. The extremely small difference is mainly on the flat rate. Due to this difference, the maximum deviations in the latitude and the altitude are 0.105 mm, respectively, and the maximum deviation of gravity is 0.016 × 10${}^{-8}$ m/s${}^{2}$. These deviations can be neglected under the current accuracy of measurement level. Thus, the GPS and BDS’s position data can be used directly without coordinate transformation.

### 4.2. The Unification of the Two Time Systems

There is no leap second problem since both BDS and GPS use atomic time. The two systems differ by 1356 weeks and 14 s due to the difference on the starting point. It gives the synchronization parameters between BDS and GPS in the navigation messages. However, the implementation content is unpublished. Thus, the two time systems cannot be fully synchronized directly. Alternatively, we choose a crude way by simply adding 14 s to BDT’s time, and adding an unknown variable to represent the time deviation in different systems [23].

## 5. The Positioning of BDS and GPS Using the Modified SR-UKF

Nonlinear Kalman filter can be well applied in the GNSS positioning estimation because of its characteristics in which the current state parameter is updated according to the observed value using the predictive value. The system model consists of the process model and the measurement model.

### 5.1. Process Model

The state model includes the receiver position and velocity coordinates in CGCS2000 coordinate system, and the receiver clock bias, which is related with states and the clock drift caused by the Doppler deviation. It also includes the non-white error in each satellite channel. Thus, the overall system state has 10 fundamental states plus one shaping state for each observable channel:

(20) $$\;\begin{array}{cc} x_{t}=(X_{t},{\dot{X}}_{t}, & Y_{t},{\dot{Y}}_{t},Z_{t},{\dot{Z}}_{t},c\delta t_{t},c\dot{\delta }t_{t},c\delta t_{t}^{sys},\delta R,\varepsilon_{1t},\varepsilon_{2t}\ldots ,\varepsilon_{nt}{)}^{T} \end{array}$$

where $\left(X_{t},Y_{t},Z_{t}\right)$ is the receiver’s position coordinate in CGCS2000, $\left({\dot{X}}_{t},{\dot{Y}}_{t},{\dot{Z}}_{t}\right)$ is the receiver velocity coordinate, $c\delta t_{t},c\dot{\delta }t_{t}$ are clock bias and clock drift bias, respectively, $c\delta t_{t}^{sys}$ is the clock deviation between GPS and BDS, δR is the offset between the Doppler shift and pseudorange’s rate, $\left(\varepsilon_{1t},\varepsilon_{2t}\ldots ,\varepsilon_{nt}\right)$ is the non-white error in each satellite channel. Because the error in each satellite is independent, it can be modeled as a first-order Gaussian–Markov process.

Since there is a deviation in the two systems’ times, in order to eliminate positioning error caused by time deviation between BDS and GPS, a variable $c\delta t_{t}^{sys}$ should be added.

Considering the above state model, and a generic kinematics model for the receiver coordinates, we obtain the associated system model:

(21) $$\;x_{t+1}=F\cdot x_{t}+C\cdot w_{t}$$

The state transition matrix *F* is given by

$$\;F=\left(\begin{array}{cc} A & 0\\ 0 & B \end{array}\right)$$

where *A* is a 10×10 time invariant matrix and *B* is a n×n diagonal matrix, which are given respectively by:

$$\;A=\left(\begin{array}{cccccccccc} 1 & T & 0 & 0 & 0 & 0 & 0 & 0 & 0 & 0\\ 0 & 1 & 0 & 0 & 0 & 0 & 0 & 0 & 0 & 0\\ 0 & 0 & 1 & T & 0 & 0 & 0 & 0 & 0 & 0\\ 0 & 0 & 0 & 1 & 0 & 0 & 0 & 0 & 0 & 0\\ 0 & 0 & 0 & 0 & 1 & T & 0 & 0 & 0 & 0\\ 0 & 0 & 0 & 0 & 0 & 1 & 0 & 0 & 0 & 0\\ 0 & 0 & 0 & 0 & 0 & 0 & 1 & T & 0 & 0\\ 0 & 0 & 0 & 0 & 0 & 0 & 0 & 1 & 0 & 0\\ 0 & 0 & 0 & 0 & 0 & 0 & 0 & 0 & 1 & 0\\ 0 & 0 & 0 & 0 & 0 & 0 & 0 & 0 & 0 & 1 \end{array}\right)$$

B=diag(α,α,⋯,α)

where $\alpha =\frac{2\tau -1}{2\tau +1},\tau =100$.

$\omega_{t}$ is the system driving noise, which is given by

(22) $$\;\begin{array}{cc} \omega_{t}= & {\left[\delta {\ddot{X}}_{t},\delta {\ddot{Y}}_{t},\delta {\ddot{Z}}_{t},w_{1t},w_{2t},w_{sys},w_{\delta R},w_{c1t},w_{c2t},\cdots ,w_{cnt}\right]}^{T} \end{array}$$

where $\delta {\ddot{X}}_{t},\delta {\ddot{Y}}_{t}$ and $\delta {\ddot{Z}}_{t}$ are noises due to the receiver acceleration and other system interferences, $w_{ci}$ is the driving noise of the *i*th shaping filters of the channel, $w_{1t}$ and $w_{2t}$ are driving noise for the clock bias model, $w_{sys}$ is the noise of the clock deviation between BDS and GPS, and $w_{\delta R}$ is the noise of δR. For each channel *i* of the received satellite, the non-white component could be modeled using the first-order Gaussian–Markov process since system errors are independent.

The noise matrix *C* is given by

$$\;C=\left(\begin{array}{cc} D & 0\\ 0 & E \end{array}\right)$$

where *D* is a 10×7 time invariant matrix and *E* is a n×n diagonal matrix, which are given respectively by

$$\;D=\left(\begin{array}{ccccccc} T^{2}/2 & 0 & 0 & 0 & 0 & 0 & 0\\ T & 0 & 0 & 0 & 0 & 0 & 0\\ 0 & T^{2}/2 & 0 & 0 & 0 & 0 & 0\\ 0 & T & 0 & 0 & 0 & 0 & 0\\ 0 & 0 & T^{2}/2 & 0 & 0 & 0 & 0\\ 0 & 0 & T & 0 & 0 & 0 & 0\\ 0 & 0 & 0 & c & 0 & 0 & 0\\ 0 & 0 & 0 & 0 & c & 0 & 0\\ 0 & 0 & 0 & 0 & 0 & 1 & 0\\ 0 & 0 & 0 & 0 & 0 & 0 & 1 \end{array}\right)$$

E=diag(β,β,⋯,β)

where $\beta =\frac{2\tau }{2\tau +1},\tau =100$.

The correspondent process noise covariance matrix $Q_{t}$ is given by

(23) $$\begin{array}{cc} Q_{t} & =E\{w_{t}w_{t}^{T}\}\\ & =\left(\begin{array}{ccccc} Q_{\delta_{\ddot{X}\ddot{Y}\ddot{Z}}} & 0 & 0 & 0 & 0\\ 0 & Q_{\delta t} & 0 & 0 & 0\\ 0 & 0 & \sigma_{\delta sys}^{2} & 0 & 0\\ 0 & 0 & 0 & \sigma_{\delta R}^{2} & 0\\ 0 & 0 & 0 & 0 & Q_{ct} \end{array}\right) \end{array}$$

with $Q_{\delta_{\ddot{X}\ddot{Y}\ddot{Z}}}=diag\left(\sigma_{\ddot{X}}^{2},\sigma_{\ddot{Y}}^{2},\sigma_{\ddot{Z}}^{2}\right)\;,\text{and}\;Q_{ct}=diag\left(\sigma_{c1}^{2},\sigma_{c2}^{2},\cdots ,\sigma_{cn}^{2}\right)$, where $\sigma_{\delta sys}^{2}$ is the variances associated with the $\delta t^{sys}$, $\sigma_{\delta R}^{2}$ is the variances associated with the δR, $\sigma_{\ddot{X}}^{2},\sigma_{\ddot{Y}}^{2}$ and $\sigma_{\ddot{Z}}^{2}$ are the process noise variances associated with $\delta {\ddot{X}}_{t},\delta {\ddot{Y}}_{t}$ and $\delta {\ddot{Z}}_{t}$, $\sigma_{c1}^{2},\sigma_{c2}^{2},\cdots ,\sigma_{cn}^{2}$ are the variances associated with the shaping filters driving noises and, $Q_{\delta t}$ is defined by following equations:

$$\;Q_{\delta t}=E\{w_{\delta t}w_{\dot{\delta }t}\}=\left(\begin{array}{cc} Q_{11} & Q_{12}\\ Q_{21} & Q_{22} \end{array}\right)$$

with

(24) $$\begin{array}{ccc} \;Q_{11} & = & \frac{h_{0}}{2}T+2h_{-1}T^{2}+\frac{2}{3}\pi^{2}h_{-2}T^{3}\\ Q_{12} & = & 2h_{-1}T+\pi^{2}h_{-2}T^{2}\\ Q_{22} & = & \frac{h_{0}}{2T}+2h_{-1}+\frac{8}{3}\pi^{2}h_{-2}T\; \end{array}$$

where $h_{0}=9.4\times 10^{-20},\;h_{-1}=1.8\times 10^{-19},\;h_{-2}=3.8\times 10^{-21}$ [3,17].

### 5.2. Measurement Model

The pseudoranges and Doppler shifts form the measurements set, and the measurement equations of the BDS’s pseudorange $\rho^{b}$ and GPS’s pseudorange $\rho^{g}$ are as follows:

(25) $$\begin{array}{cc} & \rho_{it}^{b}=\left|r_{i}-r_{it}^{b}\right|+c\delta t^{b}+\varepsilon_{it}+v_{it}\\ & =\sqrt{{\left(X_{t}-X_{it}^{b}\right)}^{2}+{\left(Y_{t}-Y_{it}^{b}\right)}^{2}+{\left(Z_{t}-Z_{it}^{b}\right)}^{2}}+c\delta t^{b}+\varepsilon_{it}+v_{it}\\ & \rho_{jt}^{g}=\left|r_{j}-r_{jt}^{g}\right|+c\delta t^{g}+\varepsilon_{jt}+v_{jt}\\ & =\sqrt{{\left(X_{t}-X_{jt}^{g}\right)}^{2}+{\left(Y_{t}-Y_{jt}^{g}\right)}^{2}+{\left(Z_{t}-Z_{jt}^{g}\right)}^{2}}+c\delta t^{g}+\varepsilon_{jt}+v_{jt}\; \end{array}$$

where $X_{t},Y_{t},Z_{t}$ are the receiver position coordinates, $X_{it}^{b},Y_{it}^{b},Z_{it}^{b}$ is the *i*th BDS satellite’s coordinates, $X_{jt}^{g},Y_{jt}^{g},Z_{jt}^{g}$ is the *j*th GPS satellite’s coordinates, $\varepsilon_{it}$ is the non-white error in satellite channel *i*, and $v_{it}$ is the measurement noise of channel *i*.

Doppler shifts give information related to the receiver velocity. Doppler is also used in our formulation, modeling it as:

(26) $$\begin{array}{cc} & D_{it}=\frac{\left(X_{t}-X_{it}\right)\left({\dot{X}}_{t}-{\dot{X}}_{it}\right)+\left(Y_{t}-Y_{it}\right)\left({\dot{Y}}_{t}-{\dot{Y}}_{it}\right)+\left(Z_{t}-Z_{it}\right)\left({\dot{Z}}_{t}-{\dot{Z}}_{it}\right)}{\sqrt{{\left(X_{t}-X_{it}\right)}^{2}+{\left(Y_{t}-Y_{it}\right)}^{2}+{\left(Z_{t}-Z_{it}\right)}^{2}}}+c\dot{\delta }t+\delta R_{t} \end{array}$$

where ${\dot{X}}_{t},{\dot{Y}}_{t},{\dot{Z}}_{t}$ are velocity coordinates of receiver at time *t*; and ${\dot{X}}_{it},{\dot{Y}}_{it},{\dot{Z}}_{it}$ is a velocity coordinate of satellite *i* at time *t* .

Thus, the system measurement $z_{t}$ for *n* satellites is given by:

(27) $$\begin{array}{cc} z_{t}= & {\left[\rho_{1t}^{b},\rho_{2t}^{b},\cdots ,\rho_{n_{1}t}^{b},\rho_{1t}^{g},\rho_{2t}^{g},\cdots ,\rho_{n_{2}t}^{g},D_{1t}^{b},D_{2t}^{b},\cdots ,D_{n_{1}t}^{b},D_{1t}^{g},D_{2t}^{g},\cdots ,D_{n_{2}t}^{g}\right]}^{T} \end{array}$$

where $n=n_{1}+n_{2}$, $n_{1}$, and $n_{2}$ are the number of measured BDS and GPS satellites.

**Figure 1:**
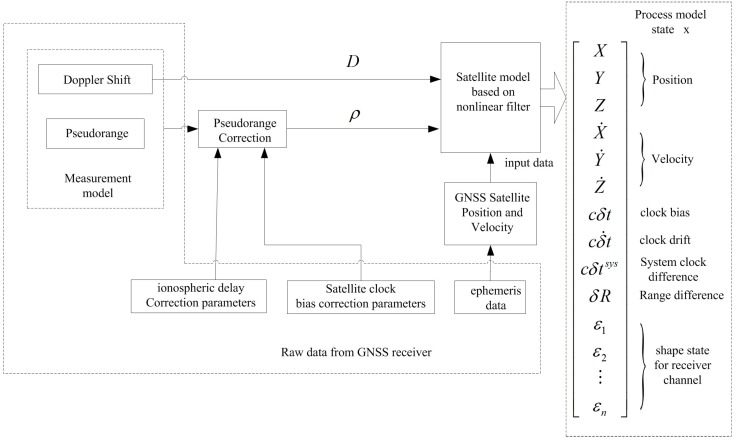
Flow of the position estimation using the nonlinear filter.

The estimation flow using the nonlinear filter is shown in Figure 1.

## 6. Experiment and Analysis

In the experiment, a set of BDS/GPS data collected by the OEM-615 (NovAtel Inc., Calgary, AB, Canada) receiver are used. This experiment was carried on School of Electronic Information and Electric Engineering of Shanghai JiaoTong University. The experimental data were collected between 7:30 a.m.–8:10 a.m., 12 October 2015. The sampling period is 1 s.

For the analysis, static receiver data were collected. The mean value of receiver’s 24 h static position data was set as the benchmark. The coordinate of the benchmark in ECEF is (-2853144.982457430,4667493.451256680,3268514.441948889).

**Figure 2:**
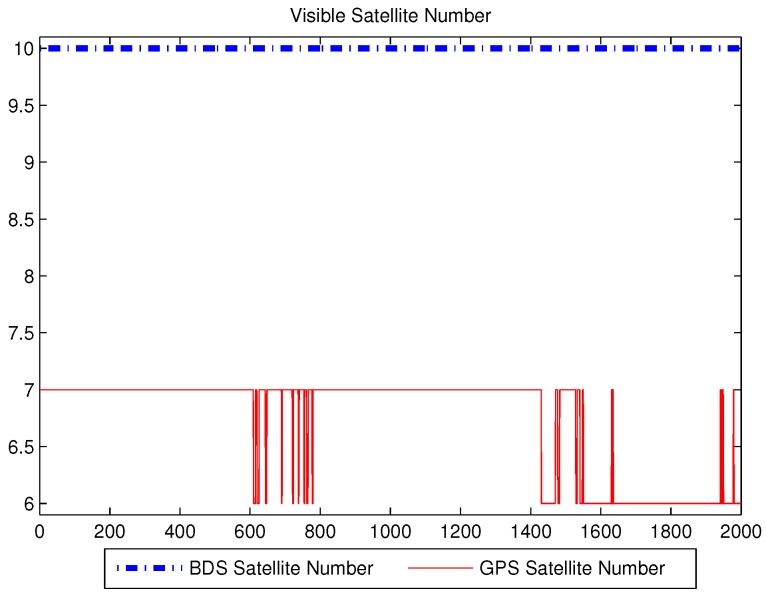
The number of visible satellites.

The number of visible satellites during the experiment’s period is shown in Figure 2. Since the observation environment was good in the outdoor with a clear view of the sky, the number of BDS satellites was stable at 10, GPS satellite numbers changed between six and seven, especially the changes in 600–800 s and 1400–1600 s were more variable. The position estimation is performed using MATLAB in the PC. The raw data was processed using four algorithms: ILS, UKF, SR-UKF and the modified SR-UKF.

**Figure 3:**
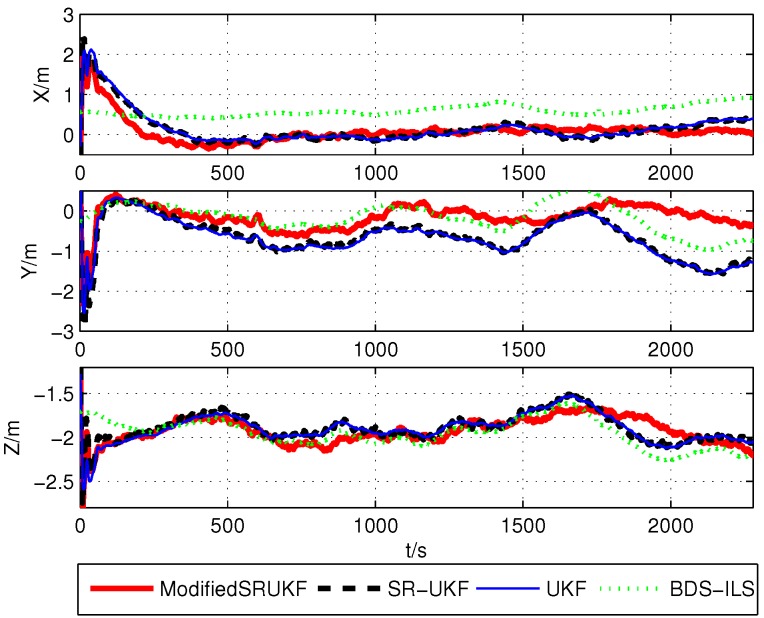
Position estimation error using BDS’s data.

**Figure 4:**
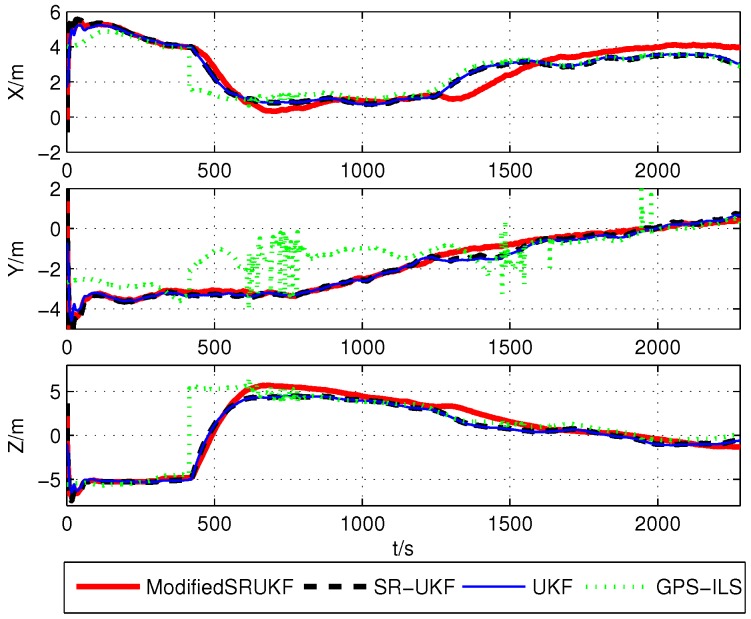
Position estimation error using GPS’s data.

**Figure 5:**
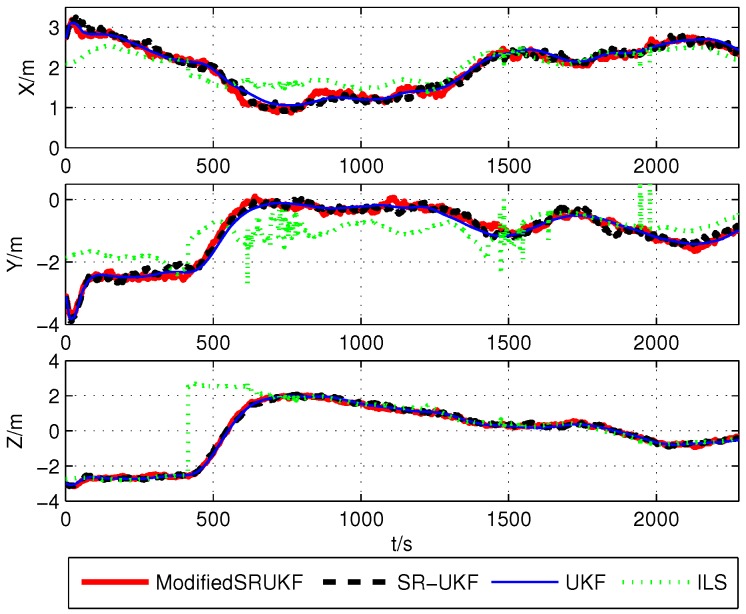
Position estimation error using BDS/GPS’s data.

The position estimation errors (x,y,z) are shown in Figure 3, Figure 4 and Figure 5.

The current BDS satellites are distributed in geostationary orbit (GEO) and inclined geosynchronous satellite orbit (IGSO) over China. That is why the BDS satellite number that is received is stable at the present stage. Due to the change in satellite numbers, the positioning results of only using GPS system experiments are not better than BDS single system and BDS/GPS dual systems.

The correspondent root mean square errors (RMSE) are compared in Table 3, Table 4 and Table 5. It is not difficult to find whether BDS, GPS or BDS/GPS positioning, the result of the modified UKF has higher accuracy than ILS, UKF and SR-UKF.

When the number of the visible satellites is large, this method cannot fully represent its superiority. We have eliminated some of the original data so as to simulate the condition of less visible satellites, the case when the numbers of BDS and GPS were in the 2–3 range.

**Figure 6:**
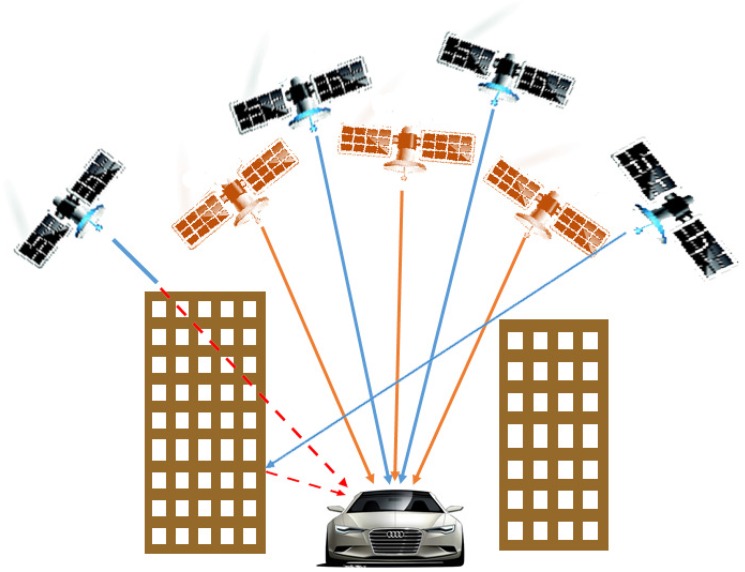
Positioning with GPS and BDS under bad conditions.

The positioning with BDS and GPS in bad environments is shown as Figure 6.

**Figure 7:**
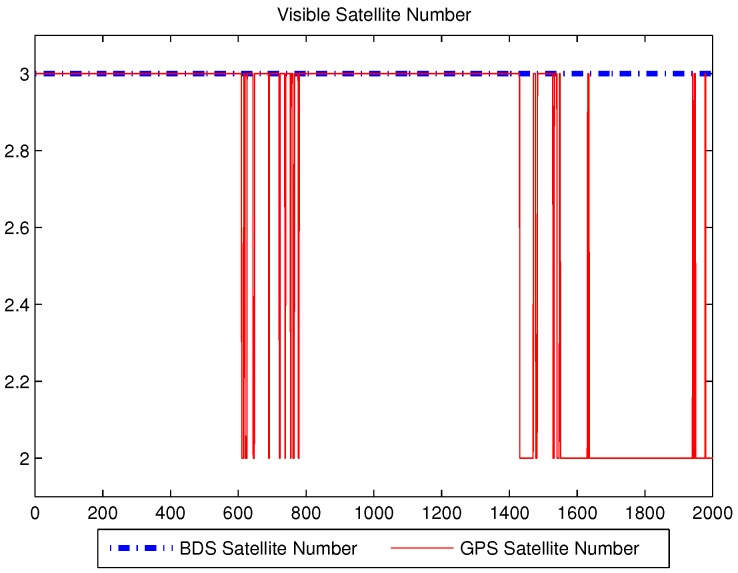
The number of visible satellites after removes.

In a single system, where there are less than four visible satellites, the positioning cannot be obtained by the conventional method, but a multi-system positioning model is a good way to deal with this situation. The number of visible satellites after part of them have been removed is shown in Figure 7. The number of the satellites of BDS remains at three, but that of GPS changes in 2–3.

When the number of satellites is less than four, the single system cannot be calculated; the advantage of multi-system positioning can now be represented. As long as the sum of BDS and GPS satellites is not less than five, the multi-system can be calculated effectively.

**Figure 8:**
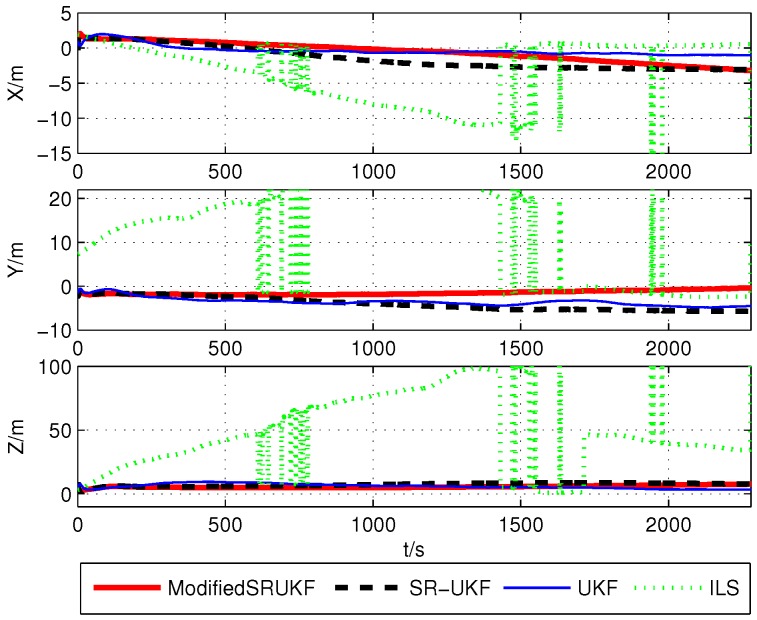
Estimation error using BDS/GPS’s Data after removal.

In Figure 8, we can see that the number of satellites is going down, ILS algorithm’s positioning precision is getting worse, and the rapid change in the number of satellites causes unsatisfactory results. Kalman filtering overcomes these difficulties well. Reduction in the number of satellites does not have a significant impact on the positioning result.

The correspondent root mean square errors of the various methods are compared in Table 6. It is not difficult to find that the result of the modified UKF has the highest accuracy. When the number of BDS or GPS satellites is too few to calculate in each single positioning system, we can adopt the multi-system positioning method. We can get the results by using the ILS algorithm, but the error is too large and it does not meet the requirements of practical application. The Kalman filtering algorithm of the multi-system can still maintain high precision, and it has a huge advantage.

## 7. Conclusions

This paper has presented the results of a study about the modified SR-UKF algorithm for multi system positioning, which was verified by the real BDS/GPS data. The proposed method is suited to the standalone GNSS positioning with low-cost and wide application. The main contributions are summarized as follows:

1. The new nonlinear positioning models for the two navigation systems were improved. Considering the differences in dual systems, a new set up for the state variables was proposed. Because there is a deviation in the two systems’ time, in order to eliminate positioning error caused by time deviation between BDS and GPS, a variable must be added. To remedy the shortage that various errors are simply taken as the Gaussian white noise in the iterative least square method, a multi-GNSS positioning model based on the nonlinear filtering was designed by establishing a suitable error model in the positioning system model and taking the nonlinear pseudorange and Doppler observation equation as the measurement equation.
2. After analyzing the characteristics of BDS and GPS, the unity of the coordinate and time systems of the two systems was considered. The BDT was selected as the time standard, and the CGCS2000 as the coordinate standard.
3. To reduce the bad effect of the old measurement data on the filtering and increase the weight of the new measurement data, the modified SR-UKF algorithm was used. The proposed algorithm gradually decreases the weighting on the old measurement data and increases it on the new measurement data, correspondingly, which overcomes the filter divergence effectively.
4. The new model and method were processed by ILS, UKF, SR-UKF and the proposed method. The experimental results show that the BDS/GPS systems positioning estimated by the proposed algorithm performs the best.
5. The proposed method also can be used in multi-system positioning in urban canyon environments. In a single system when there are less than four visible satellites, the positioning cannot be obtained by the conventional method. However, using the proposed nonlinear positioning models, the high precision positioning can be solved as long as the sum of the satellites is no less than five in the dual system. In addition, the positioning accuracy is much higher than the result obtained by the iterative least square algorithm.

Future works include applying the new model and new method to a dynamic environment, adding more GNSS systems into the environment, and making it more stable under the bad positioning conditions in which the number of satellites is not enough. In order to achieve the balance between positioning accuracy and computational complexity, we will investigate the satellite selection problem.

## Figures and Tables

**Table 1:** Unscented Kalman filter.

Calculate sigma points	$\chi_{t-1}=\left[{\hat{x}}_{t-1}\;\;\;{\hat{x}}_{t-1}\pm \sqrt{\left(L+\lambda \right)P_{t-1}}\right]$
Time-update equations	$\chi_{t|t-1}^{*}=f\left(\chi_{t-1}\right)$
	${\hat{x}}_{t|t-1}=\sum_{i=0}^{2L}W_{i}^{\left(m\right)}\chi_{i,t|t-1}^{*}$
	$P_{t|t-1}=\sum_{i=0}^{2L}W_{i}^{\left(c\right)}\left[\chi_{i,t|t-1}^{*}-{\hat{x}}_{t|t-1}^{-}\right]\cdot {\left[\chi_{i,t|t-1}^{*}-{\hat{x}}_{t|t-1}^{-}\right]}^{T}+Q$
	$z_{t|t-1}=h\left(\chi_{t|t-1}^{*}\right)$
	${\hat{z}}_{t|t-1}=\sum_{i=0}^{2L}W_{i}^{\left(m\right)}z_{i,t|t-1}$
Measurement-update equations	$P_{z_{t}z_{t}}=\sum_{i=0}^{2L}W_{i}^{\left(c\right)}\left[z_{i,t|t-1}-{\hat{z}}_{t|t-1}\right]\cdot {\left[z_{i,t|t-1}-{\hat{z}}_{t|t-1}\right]}^{T}+R$
	$P_{x_{t}z_{t}}=\sum_{i=0}^{2L}W_{i}^{\left(c\right)}\left[\chi_{i,t|t-1}^{*}-{\hat{x}}_{t|t-1}\right]\cdot {\left[z_{i,t|t-1}-{\hat{z}}_{t|t-1}\right]}^{T}$
	$K_{t}=P_{x_{t}z_{t}}P_{z_{t}z_{t}}^{-1}$
	${\hat{x}}_{t}={\hat{x}}_{t|t-1}+K_{t}\left(z_{t}-{\hat{z}}_{t|t-1}\right)$
	$P_{t}=P_{t|t-1}-K_{t}P_{z_{t}z_{t}}K_{t}^{T}$

**Table 2:** Square-root unscented Kalman filter.

Calculate sigma points	$\chi_{t-1}=\left[{\hat{x}}_{t-1}\;\;\;{\hat{x}}_{t-1}\pm \sqrt{\left(L+\lambda \right)}S_{t-1}\right]$
Time-update equations	$\chi_{t|t-1}^{*}=f\left(\chi_{t-1}\right)$
	${\hat{x}}_{t|t-1}=\sum_{i=0}^{2L}W_{i}^{\left(m\right)}\chi_{i,t|t-1}^{*}$
	$S_{t|t-1}=qr\left\{\sqrt{W_{1}^{\left(c\right)}}\left(\chi_{1:2n,t|t-1}^{*}-{\hat{x}}_{t|t-1}\right)\;\;\;\sqrt{Q}\right\}$
	$S_{t|t-1}=cholupdate\left\{S_{t|t-1},\chi_{0,t|t-1}^{*}-{\hat{x}}_{t|t-1},W_{0}^{\left(c\right)}\right\}$
	$z_{t|t-1}=h\left(\chi_{t|t-1}^{*}\right)$
	${\hat{z}}_{t|t-1}=\sum_{i=0}^{2L}W_{i}^{\left(m\right)}z_{i,t|t-1}$
Measurement-update equations	$S_{z_{t}}=qr\left\{\sqrt{W_{1}^{\left(c\right)}}\left(z_{1:2n,t|t-1}-{\hat{z}}_{t|t-1}\right)\;\;\;\sqrt{R}\right\}$
	$S_{z_{t}}=cholupdate\left\{S_{z_{t}},z_{0,t|t-1}-{\hat{z}}_{t|t-1},W_{0}^{c}\right\}$
	$P_{x_{t}z_{t}}=\sum_{i=0}^{2L}W_{i}^{\left(c\right)}\left[\chi_{i,t|t-1}^{*}-{\hat{x}}_{t|t-1}\right]\cdot {\left[z_{i,t|t-1}-{\hat{z}}_{t|t-1}\right]}^{T}$
	$K_{t}=\left(P_{x_{t}z_{t}}/S_{z_{t}}^{T}\right)/S_{z_{t}}$
	${\hat{x}}_{t}={\hat{x}}_{t|t-1}+K_{t}\left(z_{t}-{\hat{z}}_{t|t-1}\right)$
	$S_{t}=cholupdate\left\{S_{t|t-1},K_{t}S_{z_{t}},-1\right\}$

**Table 3:** Position estimation error using BDS’s data.

	RMSE /m			
	X	Y	Z	3D
ILS	0.608	0.595	2.159	2.321
UKF	0.435	0.819	1.929	2.141
SR-UKF	0.434	0.721	1.907	2.085
Modified SR-UKF	0.336	0.357	1.894	1.956

**Table 4:** Position estimation error using GPS’s data.

	RMSE /m			
	X	Y	Z	3D
ILS	3.136	2.371	3.666	5.376
UKF	3.029	2.342	3.592	5.251
SR-UKF	3.015	2.301	3.314	5.036
Modified SR-UKF	2.922	2.118	3.267	4.869

**Table 5:** Position estimation error using BDS/GPS’s data.

	RMSE /m			
	X	Y	Z	3D
ILS	2.049	1.384	1.791	3.053
UKF	2.107	1.355	1.551	2.946
SR-UKF	2.106	1.288	1.543	2.911
Modified SR-UKF	2.062	1.268	1.539	2.869

**Table 6:** Positioning estimation error using BDS/GPS’s data after removal.

	RMSE /m			
	X	Y	Z	3D
ILS	5.425	15.954	56.836	59.282
UKF	2.164	4.262	7.425	8.829
SR-UKF	1.475	3.642	6.282	7.409
Modified SR-UKF	0.801	1.567	5.731	5.995
